# Therapeutic potential of stromal cells of non-renal or renal origin in experimental chronic kidney disease

**DOI:** 10.1186/s13287-018-0960-8

**Published:** 2018-08-14

**Authors:** Cinzia Rota, Marina Morigi, Domenico Cerullo, Martino Introna, Ornella Colpani, Daniela Corna, Chiara Capelli, Ton J. Rabelink, Danielle G. Leuning, Daniela Rottoli, Ariela Benigni, Carlamaria Zoja, Giuseppe Remuzzi

**Affiliations:** 1 Istituto di Ricerche Farmacologiche Mario Negri IRCCS, Centro Anna Maria Astori, Science and Technology Park Kilometro Rosso, Via Stezzano 87, 24126 Bergamo, Italy; 2 Laboratory of Cell Therapy “G. Lanzani”, Azienda Socio Sanitaria Territoriale (ASST) Papa Giovanni XXIII, Bergamo, Italy; 3 Department of Internal Medicine, Leiden University Medical Centre, Leiden, The Netherlands; 4 Unit of Nephrology and Dialysis, Azienda Socio Sanitaria Territoriale (ASST) Papa Giovanni XXIII, Bergamo, Italy; 5 “L. Sacco” Department of Biomedical and Clinical Science, University of Milan, Milan, Italy

**Keywords:** *Mesenchymal stromal cell* therapy, Renal perivascular cells, Conditioned medium, Renal repair, Chronic kidney disease

## Abstract

**Background:**

Mesenchymal stromal cell (MSC)-based therapy is a promising strategy for preventing the progression of chronic kidney disease (CKD), with the potential to induce tissue regeneration. In search of the best cellular source we compared, in the rat model of adriamycin (ADR) nephropathy, the regenerative potential of human stromal cells of non-renal origin, such as bone marrow (bm) MSCs and umbilical cord (uc) MSCs, with that of newly discovered stromal cells of renal origin, the kidney perivascular cells (kPSCs) known to exhibit tissue-specific properties.

**Methods:**

The therapeutic effect of repeated infusions of human bmMSCs, ucMSCs, kPSCs (1.5 × 10^6^ cells/rats) or conditioned medium from ucMSCs was studied in athymic rats with ADR-induced nephropathy (7.9 mg/kg). The ability of the three stromal cell populations to engraft the damaged kidney was evaluated by detecting the presence of human nuclear antigen^pos^ cells. Glomerular podocyte loss and endothelial damage, sclerotic lesions and inflammation were assessed at 14 and 28 days. In-vitro experiments with a transwell system were performed to investigate the effects of different stromal cell populations on parietal epithelial cells (PECs) activated or not with albumin or angiotensin II for 24 h.

**Results:**

Infusions of non-renal and renal stromal cells resulted in a comparable engraftment into the kidney, in the peritubular areas and around the glomerular structures. All three cell populations limited podocyte loss and glomerular endothelial cell injury, and attenuated the formation of podocyte and PEC bridges. This translated into a reduction of glomerulosclerosis and fibrosis. Human ucMSCs had an anti-inflammatory effect superior to that of the other stromal cells, reducing macrophage infiltration and inducing polarisation towards the M2 macrophage phenotype. Conditioned medium from ucMSCs shared the same renoprotective effects of the cells. Consistent with in-vivo data, bmMSCs and kPSCs, but even more so ucMSCs, limited proliferation, migratory potential and extracellular matrix production of activated PECs, when cultured in a transwell system.

**Conclusions:**

Our data indicate that either non-renal or renal stromal cells induce renal tissue repair, highlighting ucMSCs and their conditioned medium as the most reliable clinical therapeutic tool for CKD patients.

**Electronic supplementary material:**

The online version of this article (10.1186/s13287-018-0960-8) contains supplementary material, which is available to authorized users.

## Background

Chronic kidney disease (CKD) is a key factor in poor health outcomes of major non-communicable diseases, which are the leading cause of death worldwide. CKD occurs in 10–11% of the adult population and can progress towards end-stage renal disease (ESRD), a condition that requires renal replacement therapy, such as kidney transplantation or dialysis [1, 2]. Renal transplantation is limited by a shortage of organs, and in the next decade the cost of dialysis will become unsustainable even in developed countries. Although considerable progress has been made in delaying the onset of CKD through inhibition of the renin–angiotensin system in a significant proportion of patients, the search for new therapeutic approaches to prevent or halt CKD progression remains a healthcare priority. In this context, cell-based therapy could be a promising strategy for treating CKD and is currently the focus of preclinical studies. Initial findings highlighted the therapeutic potential of mesenchymal stromal cells (MSCs) of bone marrow origin, in terms of their renotropism and regenerative effects in experimental acute kidney injury (AKI) [3–6]. Further studies demonstrated that the infusion of human bone marrow (bm)-derived MSCs and, even more, of human umbilical cord (uc) MSCs, ameliorated renal function and structure impairment, enhancing tubular cell repair and survival in mice with AKI [7, 8]. The finding that both MSC populations engrafted the renal tissue to a very low extent and did not incorporate into tubular structures is fully consistent with their paracrine activity to release spectra of prosurvival, anti-fibrotic and anti-inflammatory biomolecules that accelerated the process of renal repair in AKI [8, 9]. The evidence that MSCs induced kidney regeneration via proliferation of endogenous renal cells following injury suggested the intriguing possibility that the kidney might contain a “renopoietic system” with progenitors capable of replacing glomerular and tubular epithelial cells [10], opening new avenues of investigation in regenerative medicine. In this context, putative renal progenitor cells were isolated and characterised, and when injected into animals with AKI they were able to improve renal function and kidney tissue integrity [11–13]. More recently, a new population of perivascular stromal cells has been isolated from the human kidney (kPSCs) [14]. This cell population shares strong transcriptional similarities and common stromal cell markers with human bmMSCs, although kPSCs show renal tissue-specific expression signatures, including HoxD10 and HoxD11 transcription factors, which are crucial for nephrogenesis [14]. This tissue-specific imprinting renders kPSCs functionally different and more effective compared to bmMSCs, in terms of growth factor secretion, renal integration and survival in the neonatal kidney, and improvement of kidney injury when injected into mice with glycerol-induced AKI [14].

Despite the proven therapeutic role of mesenchymal stromal cells and renal progenitors in experimental AKI, their efficacy in slowing the progression of CKD is, at the very least, controversial [15, 16]. Preclinical studies demonstrated that bmMSC-based therapy exerted protective effects in limiting glomerular microvessel rarefaction, fibrosis and glomerulosclerosis, but failed to reduce proteinuria in different models of CKD [17–19]. Interestingly, human glomerular progenitor cells, which express mesenchymal stromal cell markers, integrated into glomerular structures acquiring phenotypic features of podocytes, and limited proteinuria and delayed progressive glomerular sclerosis when infused in mice with adriamycin (ADR) nephropathy [10]. Since none of the aforementioned studies compared the therapeutic potential of stromal cell populations of non-renal and renal origin, here we investigated the renoprotective effects of human bmMSCs or ucMSCs, with respect to the renal stromal kPSCs in immunodeficient rats with ADR-induced nephropathy. Furthermore, we studied whether the potential “memory of origin” of kPSCs could imply a higher capacity to integrate into the kidney structures and to repair injured renal tissues better than cells of non-renal origin, such as bmMSCs or ucMSCs. Another important goal was to test and compare the therapeutic efficacy of stromal cell-derived conditioned medium with the effects of the corresponding cell population.

## Methods

### Human cell isolation and characterisation

#### Human stromal cells of non-renal origin

Human bmMSCs and human ucMSCs were isolated and characterised as previously described [20, 21]. Detailed methods are provided in Additional file 1: Supplementary Methods.

#### Human kidney perivascular stromal cells

Human kPSCs were isolated from human transplant-grade kidneys, discarded for surgical reasons, by using clinical-grade protocols, enzymes and products, as previously described [14]. Briefly, within 30 h after surgery, the renal artery was cannulated and the kidney was perfused with collagenase and DNAse. After approximately 30 min, the tissue was digested and the cell suspension was cultured and expanded in αMEM 1× Glutamax (Thermo Fisher Scientific Life Science, Waltham, MA, USA; http://www.thermofisher.com) containing 5% platelet lysate, glutamine and penicillin/streptomycin. At passage 1, the kPSC population was isolated using MACS on the basis of NG2 expression [14]. Flow cytometric analysis was used to analyse the kPSC phenotype. In addition to NG2, kPSCs were positive for PDGF-R-β, CD146, CD73, CD90 and CD105, while being negative for CD31, CD34, CD45 and CD56. Osteogenic and chondrogenic, but not adipogenic, differentiation was observed in kPSCs [14].

All of the populations of stromal cells of non-renal and renal origin used for both in-vitro and in-vivo experiments were used within the fourth to sixth passage.

To prepare conditioned medium (CM), ucMSCs were incubated for 15 h in αMEM 1× Glutamax (Thermo Fisher Scientific Life Science) in serum-free conditions. Then, the medium was collected and centrifuged at 2000 × *g* for 20 min at 4 °C to remove cellular debris. After centrifugation, supernatant was transferred into Amicon Ultra-15 centrifugal Filter Devices with a 3000 molecular weight cutoff (Merck Millipore, Darmstadt, Germany; http://www.merckmillipore.com) and centrifuged at 4000 × *g* for 20 min to concentrate the volume of CM. Each aliquot of CM (500 μl) injected into ADR rats was obtained from 1.5 × 10^6^ ucMSCs.

#### Human parietal epithelial cells

Human parietal epithelial cells (PECs) were isolated and characterised as previously described [22]. Detailed methods are provided in Additional file 1: Supplementary Methods.

### In-vitro co-culture experiments

PECs were seeded at a density of 20,000 cells/cm^2^ on cover slips placed in the low chamber of a transwell system (Sigma-Aldrich, St. Louis, MO, USA; https://www.sigmaaldrich.com). One day later, the medium was replaced with experimental medium alone containing EBM (Lonza, Basel, Switzerland; http://www.lonza.com), 1% fetal bovine serum Hyclone (FBS HY; Thermo Fisher Scientific Life Science) and 1% penicillin streptomycin (PS; Thermo Fisher Scientific Life Science) with or without Angiotensin II (Ang II; 10^− 7^ M; Sigma-Aldrich) or human serum albumin (alb) (10 mg/ml; Sigma-Aldrich). After 9 h, bmMSCs, ucMSCs or kPSCs were seeded on 0.4-μm inserts (Sigma-Aldrich) at a concentration of 20,000 cells/cm^2^ in order to maintain an equal proportion with PECs. Empty inserts were also added to the wells of control PECs, PECs + Ang II or PECs + albumin to maintain the same conditions in all experimental groups. After 15 h of co-culture, inserts were removed and PECs were fixed with 2% paraformaldehyde (PFA) (Electron Microscopy Sciences, Hatfield, PA, USA; https://www.emsdiasum.com) + 4% sucrose (Sigma-Aldrich) and then used for immunofluorescence studies as already described (see Fig. 5a).

### Rat model of ADR-induced nephropathy

Male athymic rats (Hsd: RH-Foxn1rnu; Envigo RMS Srl, Udine, Italy; http://www.envigo.com), with initial body weights of 200–250 g, were used for the experiments. Animals were housed in a constant-temperature room with a 12-h dark/12-h light cycle in a specific pathogen-free facility, and fed a standard diet. Disease was induced through a single infusion of adriamycin (ADR, 7.9 mg/kg; Pfizer Italia s.r.l, Latina, Italy; http://www.pfizer.it) in the rat tail vein. Thirty-six hours after ADR treatment, rats were divided into five groups and intravenously (i.v.) injected with saline (*n* = 14), human bone marrow MSCs (1.5 × 10^6^ cells/rat/infusion, *n* = 7), human umbilical cord MSCs (1.5 × 10^6^ cells/rat/infusion, *n* = 13), human kidney perivascular stromal cells (1.5 × 10^6^ cells/rat/infusion, *n* = 7) or CM obtained from ucMSCs (CM derived from 1.5 × 10^6^ cells/rat/infusion, *n* = 6). Injections of stromal cells were repeated at different times as shown in Additional file 1: Figure S1a. Five normal rats, i.v. injected with saline, served as controls. Twenty-four-hour urine samples were collected using metabolic cages, and proteinuria was measured using the Coomassie method with a Cobas Mira autoanalyser (Roche Diagnostics System, Basel, Switzerland; http://www.roche.com). Renal function was assessed as blood urea nitrogen (BUN), in serum samples at 28 days, using the Reflotron test (Roche Diagnostics) according to the manufacturer’s instructions. Rats were sacrificed at 14 or 28 days after ADR injection, and the kidneys were removed for histology and immunohistochemistry analyses. Other organs such as the heart, liver and lung were collected for immunohistochemistry analyses. Detailed methods and a list of antibodies used for human stromal cell engraftment, renal morphology and immunohistochemistry are provided in Additional file 1: Supplementary Methods.

### Immunofluorescence studies in vitro

Detailed methods and a list of antibodies used to study PEC activation are provided in Additional file 1: Supplementary Methods.

### Statistical analysis

Results are expressed as mean ± SE. Data were analysed by ANOVA test coupled with Tukey or Dunnet post-hoc analysis, as appropriate. The statistical significance level was defined as *p* < 0.05. Data analysis was performed using GraphPad Prism (GraphPad Prism Software, Inc., La Jolla, CA, USA; https://graphpad.com).

## Results

### In-vivo studies

#### Engraftment of non-renal and renal stromal cells into the kidney of rats with ADR nephropathy

**Fig. 1:**
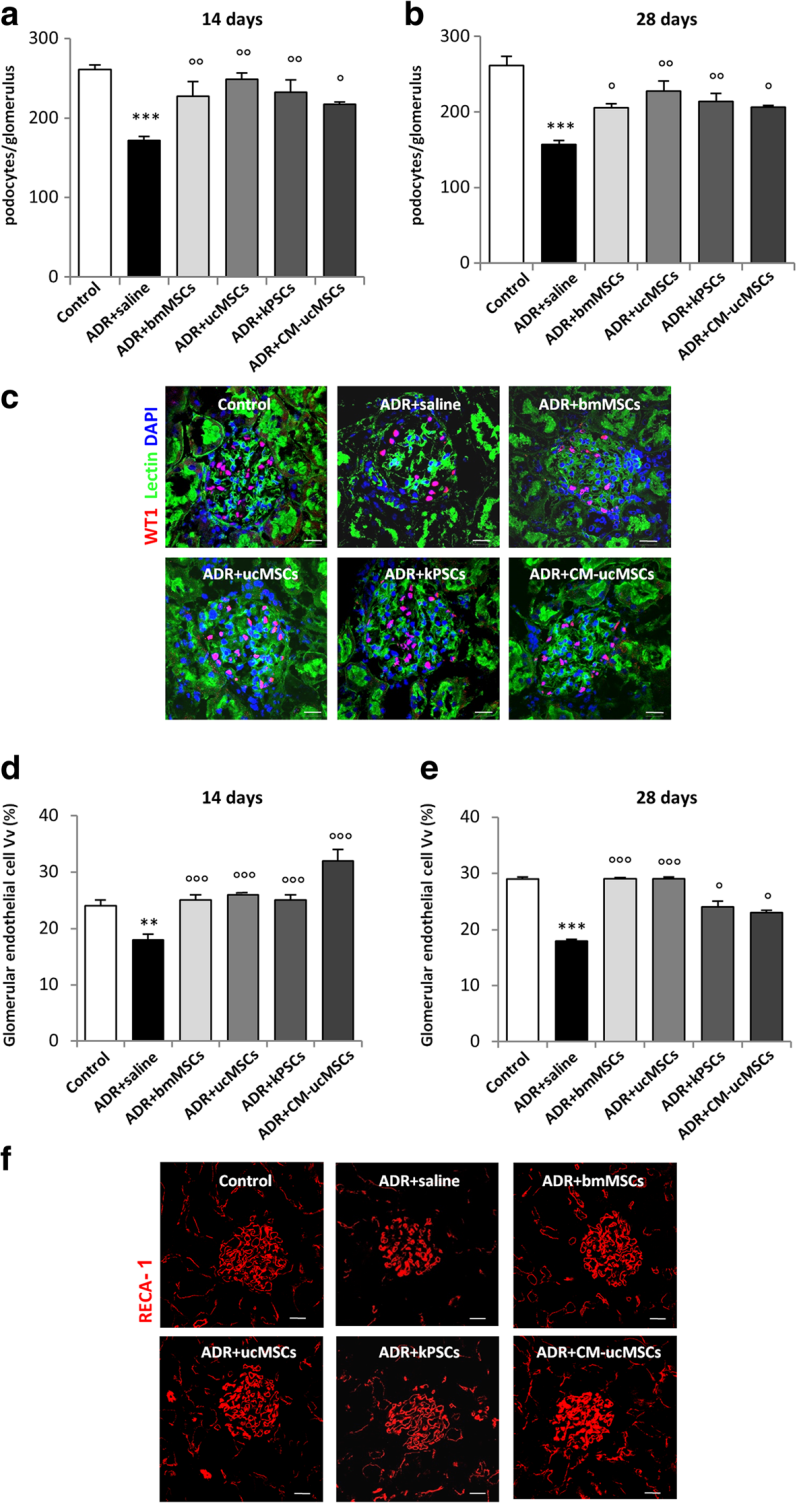
Human bmMSCs, ucMSCs, kPSCs or CM-ucMSCs reduce glomerular podocyte and endothelial cell injury in ADR rats. **a, b** Quantification of podocyte number by morphometric analysis of WT1-positive podocytes in control and ADR rats receiving saline, bmMSCs, ucMSCs, kPSCs or CM-ucMSCs at 14 days (**a**) and 28 days (**b**). Data are mean ± SE. ***p < 0.001 vs control; °*p* < 0.05 and °°*p* < 0.01 vs ADR + saline. **c** Representative micrographs of renal sections from control and ADR rats receiving saline, bmMSCs, ucMSCs, kPSCs or CM-ucMSCs showing WT-1-positive podocytes (red) at 14 days. Scale bar 20 μm. **d, e** Quantification of capillary volume density (Vv) expressed as percentage of rat endothelial cell antigen-1 (RECA-1)-positive glomerular endothelial cells in control and ADR rats receiving saline, bmMSCs, ucMSCs, kPSCs or CM-ucMSCs at 14 days (**d**) and 28 days (**e**). Data are mean ± SE. **p < 0.01 and ***p < 0.001 vs control; °*p* < 0.05 and °°°*p* < 0.001 vs ADR + saline. **f** Representative images of glomerular endothelial cells stained with RECA-1 in renal sections of control and ADR rats receiving saline, bmMSCs, ucMSCs, kPSCs or CM-ucMSCs at 14 days. Scale bar 20 μm. ADR adriamycin, bmMSC bone marrow-derived mesenchymal stromal cell, CM-ucMSC conditioned medium obtained from umbilical cord-derived mesenchymal stromal cell, DAPI 4′,6-diamidino-2-phenylindole, kPSC kidney perivascular stromal cell

**Fig. 2:**
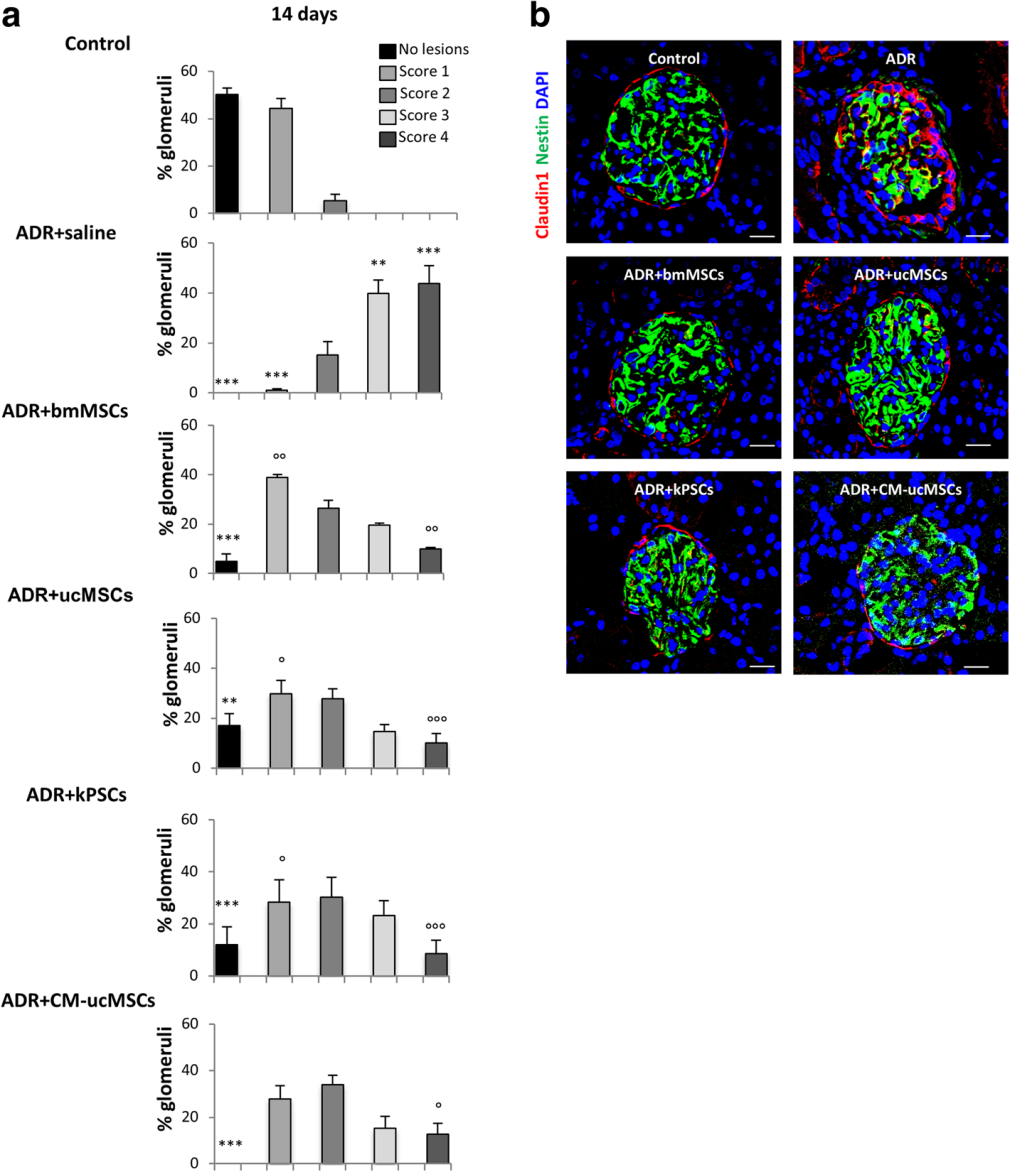
Treatment with human bmMSCs, ucMSCs, kPSCs or CM-ucMSCs reduces synechiae formation. **a** Percentage of glomeruli affected by different degrees of synechiae (intercellular podocyte–PEC bridges) in control and ADR rats receiving saline, bmMSCs, ucMSCs, kPSCs or CM-ucMSCs at 14 days. To evaluate synechiae formation, each glomerulus was assigned a score between 0 (no lesion) and 4; values expressed as mean percentage ± SE. **p < 0.01 and ***p < 0.001 vs control; °*p* < 0.05, °°*p* < 0.01 and °°°*p* < 0.001 vs ADR + saline at the corresponding score. **b** Representative images of PECs and podocytes stained with claudin 1 (red) and nestin (green), respectively, in renal sections of control and ADR rats receiving saline, bmMSCs, ucMSCs, kPSCs or CM-ucMSCs at 14 days. Nuclei stained with DAPI (blue). Scale bar 20 μm. ADR adriamycin, bmMSC bone marrow-derived mesenchymal stromal cell, CM-ucMSC conditioned medium obtained from umbilical cord-derived mesenchymal stromal cell, DAPI 4′,6-diamidino-2-phenylindole, kPSC kidney perivascular stromal cell

**Fig. 3:**
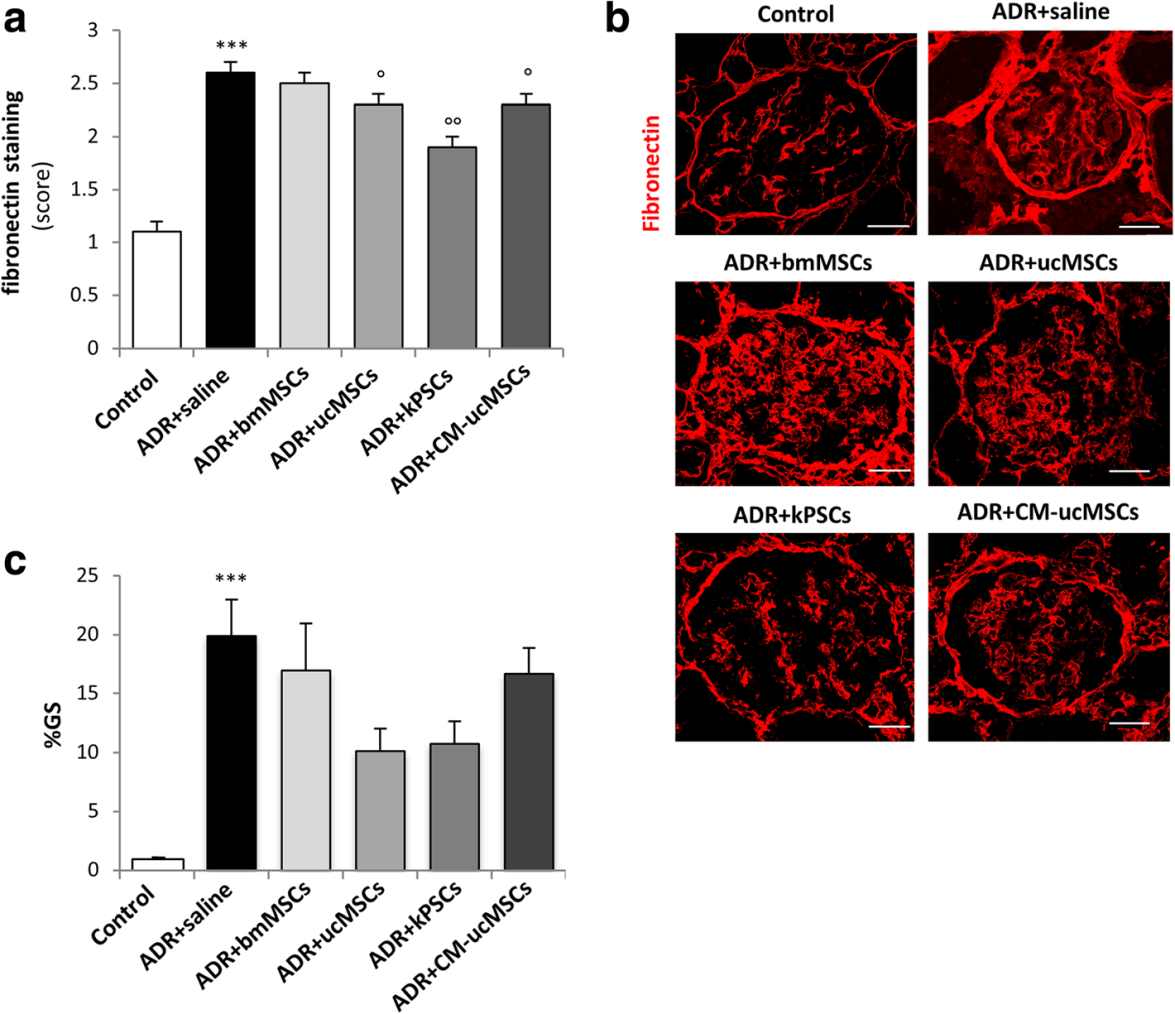
Effect of human bmMSC, ucMSC, kPSC or CM-ucMSC infusions on renal fibrosis and glomerulosclerosis. **a** Quantification of fibronectin deposits along Bowman’s capsule in control and ADR rats receiving saline, bmMSCs, ucMSCs, kPSCs or CM-ucMSCs at 28 days. Score between 0 and 3 assigned by evaluating thickness of Bowman’s capsule. Data are mean ± SE. ***p < 0.001 vs control; °*p* < 0.05 and °°*p* < 0.01 vs ADR + saline. **b** Representative images showing fibronectin expression in renal sections of control and ADR rats receiving saline, bmMSCs, ucMSCs, kPSCs or CM-ucMSCs at 28 days. Scale bar 20 μm. **c** Quantification of glomeruli affected by sclerotic lesions (%GS) assessed in PAS-stained kidney sections of control and ADR rats receiving saline, bmMSCs, ucMSCs, kPSCs or CM-ucMSCs at 28 days. Data are mean ± SE. ***p < 0.001 vs control. ADR adriamycin, bmMSC bone marrow-derived mesenchymal stromal cell, CM-ucMSC conditioned medium obtained from umbilical cord-derived mesenchymal stromal cell, kPSC kidney perivascular stromal cell

The efficacy of cell-based therapies with stromal cells of non-renal and renal origin was investigated by comparing the renoprotective effects of bmMSCs and ucMSCs with kPSCs in immunocompromised rats with ADR-induced nephropathy, a model mimicking focal segmental glomerulosclerosis [23]. At 7 days, athymic rats injected with ADR exhibited proteinuria, which increased progressively over time (Table 1). Proteinuria was associated with glomerular podocyte and endothelial cell damage and the formation of synechiae, resulting in glomerulosclerotic lesions at 28 days after ADR injection (Figs. 1, 2, and 3). At this time, renal function, assessed as BUN, was within the normal range (Additional file 1: Table S1) .

**Table 1:** Time course of urinary protein excretion in control and ADR rats receiving saline, bmMSCs, ucMSCs, kPSCs or CM-ucMSCs

	Proteinuria (mg/day)				
	0 days	7 days	14 days	21 days	28 days
Control	6 ± 1.1	10 ± 1.3	12 ± 1.2	13 ± 1.3	13 ± 1.7
ADR + saline	7 ± 0.6	71 ± 7*	344 ± 29*	528 ± 26*	501 ± 53*
ADR + bmMSCs	7 ± 0.7	59 ± 12*	270 ± 19*	411 ± 17*	526 ± 36*
ADR + ucMSCs	7 ± 0.6	71 ± 11*	345 ± 16*	476 ± 15*	512 ± 43*
ADR + kPSCs	7 ± 0.4	63 ± 11*	355 ± 28*	471 ± 52*	486 ± 38*
ADR + CM-ucMSCs	8 ± 0.6	73 ± 13*	366 ± 40*	527 ± 37*	454 ± 48*

Data presented as mean ± standard error

*ADR* adriamycin, *bmMSC* bone marrow mesenchymal stromal cell, *ucMSC* umbilical cord mesenchymal stromal cell, *kPSC* kidney perivascular stromal cell, *CM-ucMSC* conditioned medium obtained from umbilical cord mesenchymal stromal cell

*p < 0.001 vs control at the corresponding time. Analysis of variance corrected with Tukey coefficient

The ability of human bmMSCs or ucMSCs to migrate into the damaged kidney was compared with that of kPSCs, the stromal cell population that exhibits renal tissue-specific imprinting [14]. Because of the severity of the disease, ADR rats received six infusions of non-renal or renal stromal cells in order to maintain, virtually, a constant number of engrafted cells in the injured kidneys throughout the course of the disease (Additional file 1: Figure S1a). Quantification of the intra-renal recruitment of human cells, positive for the human nuclear antigen (HNA), in ADR rats at day 14 showed that bmMSCs engrafting the kidney averaged 2.5 ± 0.4/10^5^ renal cells, ucMSCs averaged 3.1 ± 0.4/10^5^ renal cells and kPSCs averaged 3.2 ± 0.8/10^5^ renal cells. Similar renal engraftment was detected when human cells were labelled with the anti-human mitochondria antibody (bmMSCs 3.7 ± 0.5/10^5^ renal cells, ucMSCs 3.2 ± 1.1/10^5^ renal cells and kPSCs 2.6 ± 0.5/10^5^ renal cells), thus indicating that all of the cell populations migrated into the damaged renal tissue to a similar extent. Moreover, non-renal as well as renal stromal cells predominantly localised in the peritubular areas and around the glomerular structures (Additional file 1: Figure S1b). At 14 days, the analysis of other organs such as the heart and liver showed the absence of human cell engraftment in ADR rats infused with non-renal or renal stromal cells. Conversely, rare single human stromal cells were found in the lung parenchyma.

#### Effect of non-renal and renal stromal cells on glomerular podocyte and endothelial cell injury

Since podocytes are one of the main targets of glomerular injury in ADR nephropathy [18, 19], we evaluated the effects of injections of non-renal and renal stromal cells on podocyte loss in ADR rats. Using morphometric analysis, a significant reduction in the number of WT-1-positive cells was observed in ADR rats receiving saline compared to control rats at both 14 and 28 days (35% and 40% podocyte reduction, respectively) (Fig. 1a–c). In the same animals, the expression of nephrin, a key protein of the slit diaphragm, was reduced as evidenced by glomerular staining at 14 days (nephrin expression: ADR *+* saline 2.5 ± 0.5% vs control 14.9 ± 0.7% glomerular area; *p* < 0.001) (Additional file 1: Figure S1c). Systemic delivery of bmMSCs, ucMSCs or kPSCs in ADR rats limited podocyte loss, as indicated by the significantly higher number of podocytes per glomerulus compared to ADR rats receiving saline (Fig. 1a–c). Treatment with ucMSCs partially restored nephrin expression, which was unaffected by bmMSCs or kPSCs (nephrin expression: ADR + ucMSCs 6.4 ± 0.6%, ADR + bmMSCs 2.6 ± 0.3% and ADR + kPSCs 4.3 ± 0.8% glomerular area) (Additional file 1: Figure S1c). Therapy with the three stromal cell populations did not ameliorate proteinuria (Table 1) and renal function (Additional file 1: Table S1).

In parallel to podocyte injury, a marked glomerular microvessel rarefaction, evaluated as the percentage of endothelial cell volume density (%Vv), was observed in ADR rats receiving saline at 14 and 28 days (Fig. 1d–f). Notably, at these time points, treatments with bmMSCs, ucMSCs or kPSCs significantly limited glomerular endothelial cell injury (Fig. 1d–f).

Then, we analysed whether non-renal or renal stromal cell therapies could limit glomerular podocyte and endothelial cell injury in ADR rats by preventing cell apoptosis. ADR rats receiving saline exhibited an increased number of glomerular apoptotic cells positive for cleaved caspase-3 at 14 days (Additional file 1: Figure S2a, b). Infusions of bmMSCs, ucMSCs or kPSCs significantly decreased glomerular cell apoptosis (Additional file 1: Figure S2a, b).

#### Effect of non-renal and renal stromal cells on renal fibrosis

In experimental ADR nephropathy, podocyte injury promotes PEC activation and dysfunction, which leads to the formation of synechiae and glomerulosclerotic lesions [18, 22, 24–26]*.* In our setting, intercellular adhesions between the glomerular tuft and the Bowman’s capsule with a score of ≥ 3 were found in more than 80% of glomeruli in ADR rats receiving saline at 14 days (Fig. 2a). The phenotype of cells contributing to the formation of synechiae was characterised by co-staining of claudin 1 and nestin, specific markers of PECs and podocytes, respectively. Immunofluorescence staining revealed that both glomerular cell populations participated in the formation of synechiae. In particular, thickening of the Bowman’s capsule was characterised by multiple layers of claudin-1-positive cells, which migrated towards the capillary tuft, creating cellular bridges with podocytes in the renal tissue of ADR rats at 14 days (Fig. 2b). Treatments with bmMSCs, ucMSCs or kPSCs significantly lowered the percentage of glomeruli affected by synechiae with a high score compared to that observed in ADR rats given saline, while the number of glomeruli with no or few adhesions (score 0–2) increased (Fig. 2a), as also appeared in the representative images of Fig. 2b.

Synechiae formation was followed by the development of glomerular fibrotic and sclerotic lesions. As shown in Fig. 3a, b, at 28 days we observed a significant increase in fibronectin staining along the Bowman’s capsule in the renal tissues of ADR-treated rats compared to control rats. Infusions of ucMSCs or kPSCs, but not of bmMSCs, markedly reduced fibronectin deposition. Consistently, ADR-treated rats exhibited a significant increase in the percentage of glomeruli filled by sclerotic lesions at 28 days (Fig. 3c). Glomerulosclerotic lesions were less diffuse and severe in the renal tissues of ADR rats treated with ucMSCs or kPSCs (50% and 45% reduction, respectively) compared to rats receiving saline (Fig. 3c). Since TGF-β is a potent promoter of renal fibrotic processes and its upregulation is a common feature of many forms of CKD [27], we analysed its concentration in serum samples from ADR rats. At 14 days, a marked increase in TGF-β serum levels was observed in ADR rats given saline, compared to control rats (control 78 ± 7 ng/ml vs ADR + saline 126 ± 9 ng/ml; *p* < 0.01). At this time, injections of ucMSCs and kPSCs, but not of bmMSCs, normalised serum TGF-β (ADR + bmMSCs 145 ± 9 ng/ml, ADR + ucMSCs 93 ± 10 ng/ml and ADR + kPSCs 86 ± 10 ng/ml; *p* < 0.05).

#### Effect of non-renal and renal stromal cells on monocyte/macrophage infiltration in the kidney

**Fig. 4:**
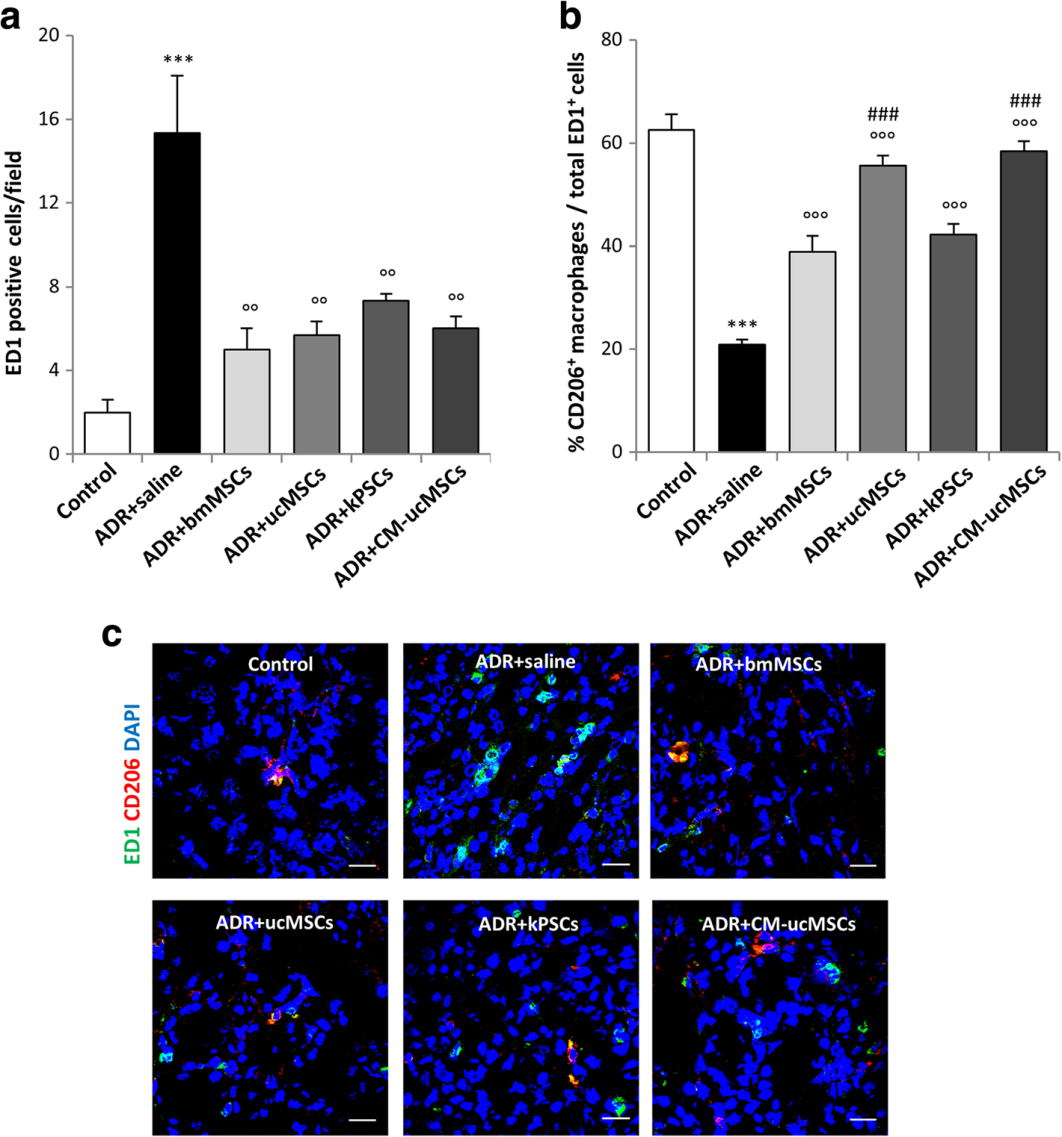
Human bmMSCs, ucMSCs, kPSCs or CM-ucMSCs decrease renal macrophage infiltration. **a** Quantification of ED1-positive monocytes/macrophages per field in renal tissue of control and ADR rats receiving saline, bmMSCs, ucMSCs, kPSCs or CM-ucMSCs at 14 days. Data are mean ± SE. ***p < 0.001 vs control; °°*p* < 0.01 vs ADR + saline. **b** Quantification of macrophages with anti-inflammatory M2 phenotype expressed as percentage of CD206-positive macrophages per total ED1-positive cells in renal sections of control and ADR rats receiving saline, bmMSCs, ucMSCs, kPSCs or CM-ucMSCs at 14 days. Data are mean ± SE. ***p < 0.001 vs control; °°°*p* < 0.001 vs ADR + saline; ###*p* < 0.001 vs ADR + saline + bmMSCs or kPSCs. **c** Representative images of ED1-positive monocytes/macrophages (green) and anti-inflammatory M2 macrophages labelled with CD206 (red) in renal tissue of control and ADR rats receiving saline, bmMSCs, ucMSCs, kPSCs or CM-ucMSCs at 14 days. Nuclei stained with DAPI (blue). Scale bar 20 μm. ADR adriamycin, bmMSC bone marrow-derived mesenchymal stromal cell, CM-ucMSC conditioned medium obtained from umbilical cord-derived mesenchymal stromal cell, DAPI 4′,6-diamidino-2-phenylindole, kPSC kidney perivascular stromal cell

We then explored the anti-inflammatory activity of bmMSCs and ucMSCs versus kPSCs in ADR nephropathy. A large number of ED1-positive monocytes/macrophages accumulated in the renal tissues of ADR rats given vehicle at 14 days (Fig. 4a). Treatment with non-renal and renal stromal cell populations had a remarkable anti-inflammatory effect, resulting in a significant reduction in the number of infiltrating monocytes/macrophages (Fig. 4a, c). Since bmMSCs are known to be able to promote the shift of macrophages from the proinflammatory M1 to the anti-inflammatory M2-like phenotype [28], we studied whether therapies with the three stromal cell populations could modulate macrophage phenotypic changes in the setting of ADR nephropathy. Co-staining of ED1-positive monocytes/macrophages with CD206, a marker highly expressed by M2 macrophages [29], revealed a significant decrease in the percentage of M2 macrophages over the totality of ED1-positive cells (Fig. 4b, c). Notably, treatment with the three stromal cell populations enhanced the percentage of M2 macrophages. In particular, ucMSCs exhibited the most potent anti-inflammatory properties (Fig. 4b, c).

#### Treatment with ucMSC-derived conditioned medium is renoprotective in rats with ADR nephropathy

Since ucMSCs appeared to be the most effective cells that exert paracrine activity on renal cells, we tested whether their conditioned medium (CM) had a therapeutic effect similar to that observed with the corresponding cells. Repeated injections of CM-ucMSCs preserved podocyte loss, compared to ADR rats treated with saline (Fig. 1a–c). No changes in nephrin expression (day 14: ADR + CM-ucMSCs 2.0 ± 0.5% vs ADR + saline 2.5 ± 0.5% glomerular area) (Additional file 1: Figure S1c) and proteinuria (Table 1) were observed. Treatment with CM significantly limited glomerular microvessel rarefaction by restoring endothelial cell volume density at both 14 and 28 days (Fig. 1d–f). Moreover, ADR rats infused with CM showed a remarkable decrease in glomerular apoptotic cells positive for cleaved caspase-3 at 14 days (Additional file 1: Figure S2a, b).

When we looked at synechiae formation, we observed that the percentage of damaged glomeruli with a high score (score 4) was significantly lower in CM-treated ADR rats, which exhibited a normal glomerular podocyte and PEC distribution (Fig. 2a, b). In addition, reduced fibronectin deposition along the Bowman’s capsule and decreased glomerulosclerosis were consistently observed in ADR rats in response to CM therapy (Fig. 3a–c). The beneficial effect of CM on fibrotic lesions was associated with the reduced serum levels of TGF-β at 14 days (ADR + CM-ucMSCs 83 ± 4 ng/ml vs ADR + saline 126 ± 9 ng/ml; *p* < 0.01). CM had a significant anti-inflammatory effect, as demonstrated by a lower number of infiltrated ED1 macrophages and a significant increase of M2 macrophages in the renal tissues of ADR rats (Fig. 4a–c). These data indicate that treatment with conditioned medium shared the renoprotective effects observed with ucMSC therapy.

### In-vitro studies

**Fig. 5:**
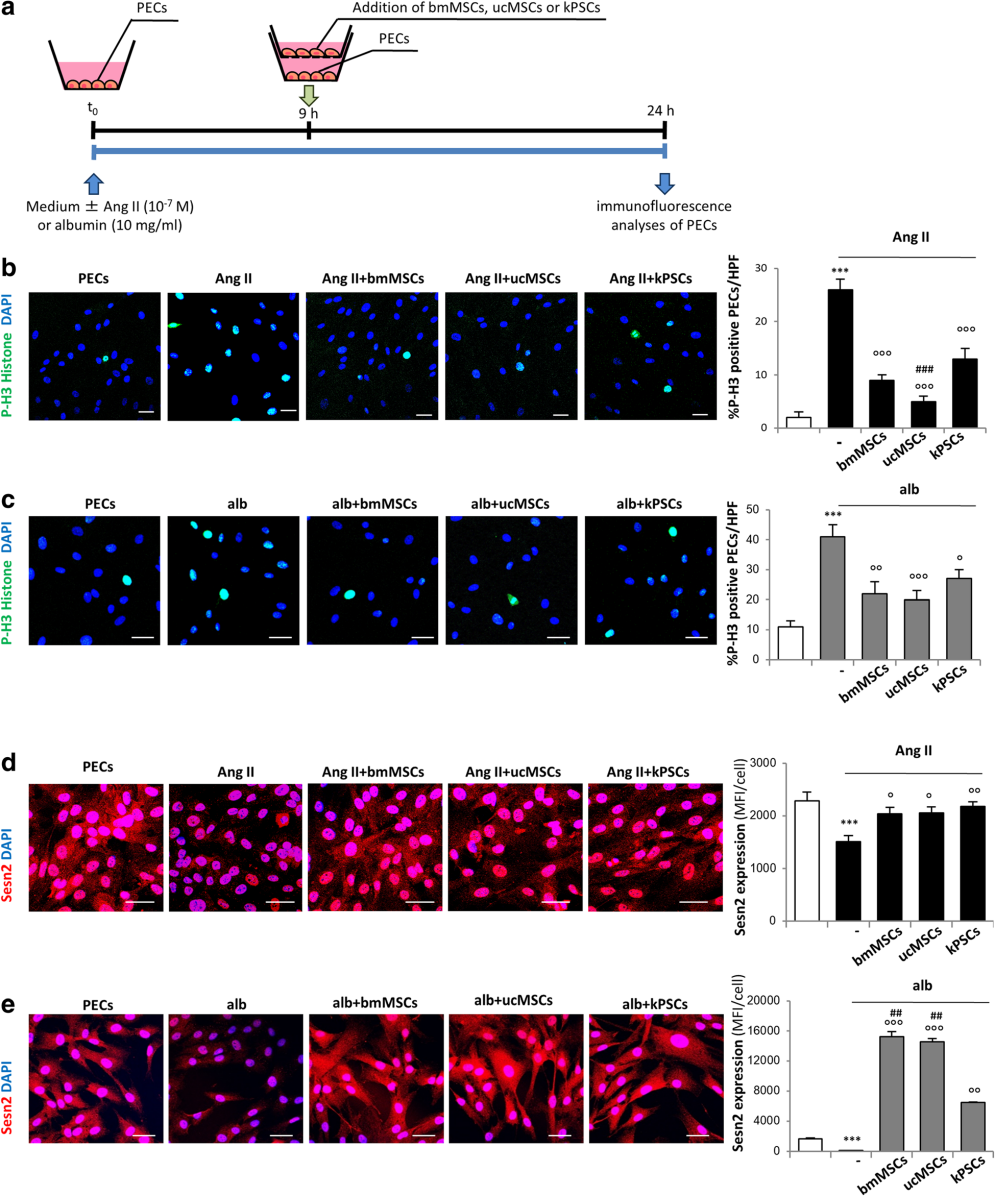
Effect of human bmMSCs, ucMSCs or kPSCs on proliferation of activated PECs in co-culture system. **a** Schematic representation of experimental design with activated human PECs and stromal cells in co-culture using a transwell system. **b, c** Representative images and quantification of proliferating PECs positive for phospho H3-histone (P-H3) exposed to medium alone and angiotensin II (Ang II, 10^− 7^ M) (**b**) or albumin (alb, 10 mg/ml) (**c**) and co-cultured with bmMSCs, ucMSCs or kPSCs. PEC nuclei stained with DAPI. Data expressed as percentage of P-H3 positive PECs per total DAPI-positive cells/HPF. ***p < 0.001 vs PECs; °*p* < 0.05, °°*p* < 0.01 vs PECs + albumin; °°°*p* < 0.001 vs PECs + Ang II or albumin; ###*p* < 0.001 vs PECs + Ang II + kPSCs. **d, e** Representative images and quantification of Sestrin 2 (Sesn2) expression in PECs exposed to medium alone and Ang II (**d**) or alb (**e**) and co-cultured with bmMSCs, ucMSCs or kPSCs. PEC nuclei stained with DAPI (blue). Data expressed as MFI/cell. ***p < 0.001 vs PECs; °*p* < 0.05 vs PECs + Ang II; °°*p* < 0.01 vs PECs + Ang II or alb; °°°*p* < 0.001 vs PECs + alb; ##*p* < 0.01 vs PECs + alb + kPSCs. Data are mean ± SE. Scale bar 20 μm. bmMSC bone marrow-derived mesenchymal stromal cell, DAPI 4′,6-diamidino-2-phenylindole, HPF high-power field, kPSC kidney perivascular stromal cell, MFI mean fluorescence intensity, PEC parietal epithelial cell, ucMSC umbilical cord-derived mesenchymal stromal cell

To identify the molecular processes driving the recovery of the PEC phenotype in vivo in response to cell therapies, in-vitro experiments were performed using a transwell system. PECs were activated Ang II or albumin, mimicking the disease condition, and were co-cultured with bmMSCs, ucMSCs or kPSCs seeded on the insert of the transwell (Fig. 5a). PEC proliferation was studied by evaluating the number of cell nuclei positive for phospho H3-histone (P-H3), a marker of mitosis. Results showed that PECs exposed for 24 h to Ang II or albumin proliferated markedly compared to control cells (Fig. 5b, c). Co-culture of activated PECs with bmMSCs, ucMSCs or kPSCs significantly decreased PEC proliferation in response to both stimuli. Notably, ucMSCs inhibited PEC proliferation to a more significant extent than bmMSCs or kPSCs did (Fig. 5b, c). To further investigate the mechanisms regulating PEC proliferation, we studied the expression of sestrin 2 (Sesn2), a stress-inducible protein that counteracts oxidative stress and represses cell proliferation by targeting mTOR signalling [30, 31]. As shown in Fig. 5d, e, control PECs constitutively expressed Sesn2, which decreased markedly following Ang II and albumin exposure. Transwell co-culture of bmMSCs, ucMSCs or kPSCs significantly upregulated Sesn2 expression in PECs activated with both stimuli (Fig. 5d, e).

**Fig. 6:**
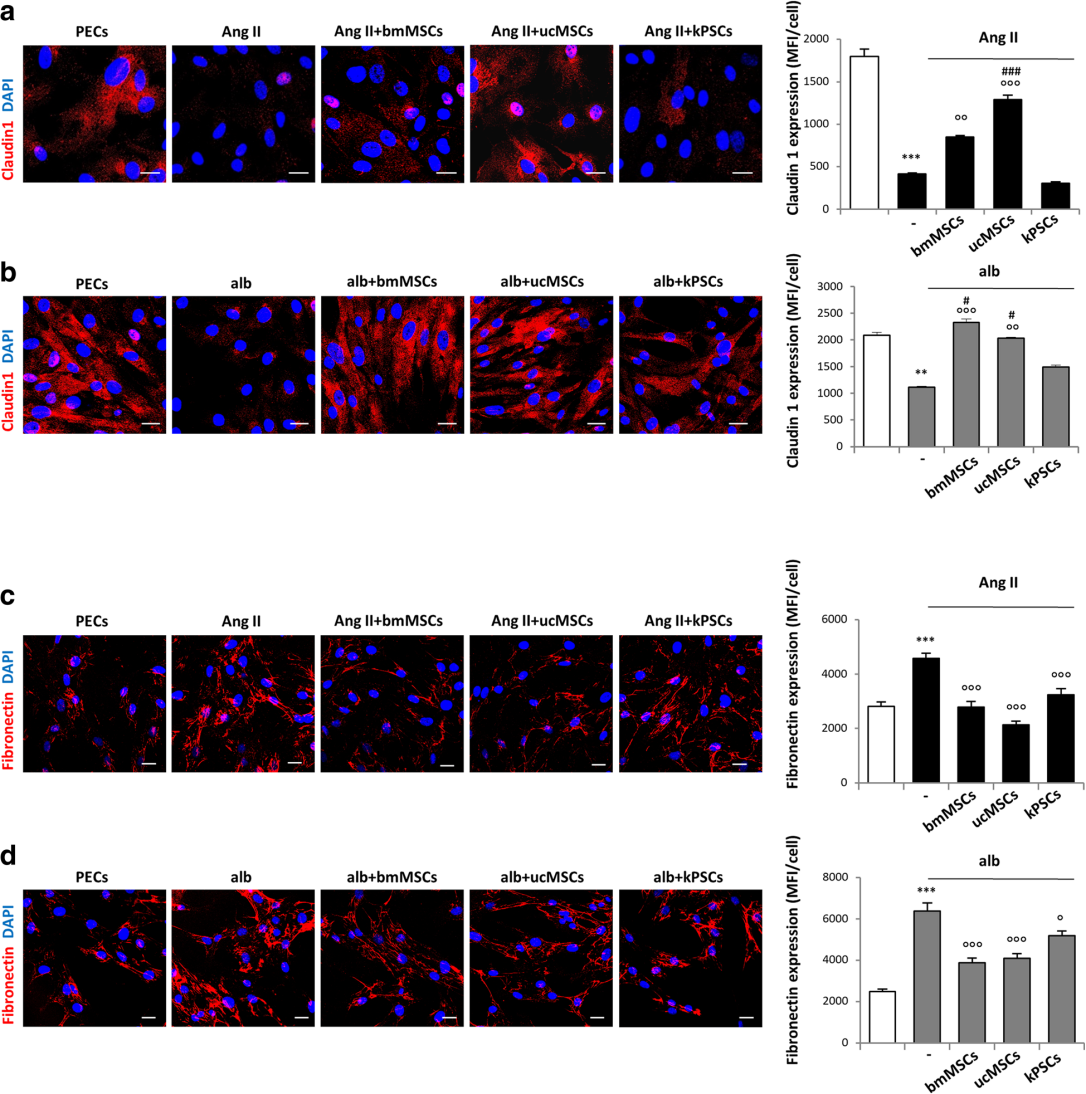
Effect of human bmMSCs, ucMSCs or kPSCs on claudin 1 and fibronectin expression in activated PECs. **a, b** Representative images and quantification of claudin 1 expression in PECs exposed to medium alone and angiotensin II (Ang II) (**a**) or albumin (alb) (**b**) and co-cultured with bmMSCs, ucMSCs or kPSCs. PEC nuclei stained with DAPI (blue). Data expressed as MFI/cell. **p < 0.01 and ***p < 0.001 vs PECs; °°*p* < 0.01 and °°°*p* < 0.001 vs PECs + Ang II or alb; #*p* < 0.05 vs PECs + alb + kPSCs; ###*p* < 0.001 vs PECs + Ang II + kPSCs. **c, d** Representative images and quantification of fibronectin (red) production by PECs exposed to medium alone and Ang II (**c**) or alb (**d**) and co-cultured with bmMSCs, ucMSCs or kPSCs for 24 h. PEC nuclei stained with DAPI (blue). Data expressed as MFI/cell. ***p < 0.001 vs PECs; °*p* < 0.05 vs PECs + alb; °°°*p* < 0.001 vs PECs + Ang II or alb. Data are mean ± SE. Scale bar 20 μm. bmMSC bone marrow-derived mesenchymal stromal cell, DAPI 4′,6-diamidino-2-phenylindole, kPSC kidney perivascular stromal cell, MFI mean fluorescence intensity, PEC parietal epithelial cell, ucMSC umbilical cord-derived mesenchymal stromal cell

Several pieces of in-vivo evidence demonstrated that activated PECs, migrating towards the glomerular capillary tuft, are important players in the development and progression of glomerular damage [22]. Since PEC migration involves the loss of cell–cell interaction along the Bowman’s capsule, we evaluated the expression of claudin 1, a tight junction protein that is constitutively expressed by PECs. Parietal epithelial cells activated by Ang II showed a significant decrease in claudin 1 expression, compared to control cells, that was restored when PECs were in co-culture with bmMSCs or ucMSCs, but not kPSCs (Fig. 6a). Notably, ucMSCs increased claudin 1 expression more efficiently in activated PECs (Fig. 6a). Similar data were obtained when PECs activated with albumin were co-cultured with stromal cells (Fig. 6b).

Next, the capacity of stromal cells to modulate extracellular matrix (ECM) production by activated PECs was explored. Both Ang II and albumin enhanced the production of fibronectin compared to control PECs (Fig. 6c, d), which was significantly reduced by co-culture with bmMSCs, ucMSCs or kPSCs (Fig. 6c, d). These in-vitro data demonstrate that the human stromal cells of non-renal and renal origin are likewise able to limit PEC dysfunction, restoring their phenotype, and propose ucMSCs as the most effective cell population.

## Discussion

This study provides novel insights into the therapeutic potential of human stromal cells of different origins, having compared the regenerative activity of non-renal and renal stromal cells in a chronic model of adriamycin (ADR)-induced nephropathy. In this setting, we also provide the proof of concept that therapy with stromal cell-derived conditioned medium mimics the renoprotective effects of the corresponding cells.

Our results demonstrate that all of the stromal cell populations tested—bmMSCs, ucMSCs or kPSCs—were able to promote renal repair by limiting podocyte loss and glomerular capillary rarefaction, ultimately leading to a reduction in PEC dysfunction. It is well established that podocyte injury is one of the first events to trigger the aberrant activation of PECs [18, 22, 32, 33] which, after losing cell–cell contact, begin to proliferate and migrate, leading to hyperplastic-like lesions, a detrimental step that drives the formation of fibrosis and glomerulosclerosis. The present data, showing that non-renal or renal stromal cells inhibit the production of profibrotic factors and minimise glomerular fibrosis and, to a lesser extent sclerotic lesions, point to dysfunctional podocytes and PECs as valuable targets for cell-based therapies, which can restore the structural integrity of glomeruli. The fact that none of the cell treatments affect proteinuria could be ascribed to the partial recovery of the podocyte slit diaphragm proteins, which are not sufficient to restore normal structure of the foot processes, as also previously described [18, 19].

Seeking to identify the mechanisms that may sustain the process of renal repair, we focused on the effects of stromal cell therapies on inflammatory cell infiltration [34], a typical feature of progressive CKD [35, 36]. Converging evidence demonstrates that macrophages play a key role in host defence and homeostatic functions, including tissue repair and the regulation of metabolic activities [37]. Depending on the local microenvironment, macrophages can acquire two opposing and competing functional phenotypes, often referred to as classical activated M1 macrophages, with proinflammatory activity, and M2 macrophages, which have anti-inflammatory and tissue repair functions, due to their peculiar secretion profile [35]. The effects of MSCs on macrophage polarisation, and their potential to alter the proinflammatory course of renal disease, remain largely unknown. Our study demonstrates that the accumulation of proinflammatory macrophages in damaged kidneys is reduced by cell treatments, documenting that either non-renal or renal stromal cells have strong anti-inflammatory activity. The picture that emerges, accompanied by evidence that M1 macrophages switch towards the M2 phenotype, suggests that cell therapies counteract renal disease outcomes by regulating macrophage polarisation, possibly through paracrine activity. These findings are consistent with previous studies, which demonstrated the capacity of bmMSCs to drive macrophage polarisation towards the M2 phenotype by producing soluble factors, including PGE2, TSG6, IL-6, IL-8, IDO and TGF-β1, following ischaemia reperfusion injury, sepsis and other disease models in which inflammation was the underlying mechanism [28, 38]. Notably, ucMSCs and their conditioned medium have stronger anti-inflammatory and renoprotective effects than the other cell populations tested, which is probably ascribable to a peculiar secretome profile of ucMSCs [39–41], rather than their distinct ability to migrate into the injured renal tissue. In this context, the finding that kPSCs exhibit a capacity to engraft into the damaged kidney that is similar to bmMSCs and ucMSCs, and that they fail to incorporate into renal structures contrary to expectation, considering that the renal microenvironment is their natural niche [14], may suggest that renal stromal cells are renoprotective through a paracrine activity like non-renal stromal cells are.

As well as producing pro-survival, anti-fibrotic and anti-inflammatory soluble factors, stromal cells can also release extracellular vesicles (EVs) which, by shuttling genetic information such as mRNAs and transcriptional regulators into target cells through specific receptor ligands, can induce cell phenotypic changes and tissue repair [42, 43]. These mechanisms of MSC–cell interaction create novel possibilities for the clinical application of stromal cell therapy in regenerative medicine, because they bypass the safety concerns and limitations associated with viable cell transplantation [44]. In our CKD setting, the renoprotective effects of conditioned medium, possibly ascribable to the presence of bioactive molecules and EVs, were consistent with the evidence describing the regenerative effects of MSC-derived EV treatment for acute and chronic renal diseases [45, 46].

It is possible to infer, as observed in vivo, that non-renal and renal stromal cells, when in a co-culture system, can communicate with glomerular cell populations through the release of soluble factors and EVs in their conditioned media, thus reducing podocyte injury [18] and PEC activation.

Indeed, bmMSCs and kPSCs, and particularly ucMSCs, can produce factors able to limit the proliferation of activated PECs, possibly by normalising the expression of Sestrin 2, an inhibitor of mTOR mitogenic signalling [31]. A previous study, which demonstrates that the decrease in Sestrin 2 expression and consequent mTOR activation in PECs is strongly associated with the development of glomerular hyperplastic-like lesions and periglomerular fibrosis in animal models of puromycin nephropathy and crescentic glomerulonephritis, supports the idea that Sestrin 2 has a critical role in regulating the PEC phenotype [31]. Interestingly, our data further show that exposing cultured activated PECs to media conditioned by non-renal and renal stromal cells restores the abnormal extracellular matrix production and the expression of claudin 1, a junctional protein regulating their migratory behaviour. These data highlight the potential of stromal cell-derived bioactive products, particularly those of ucMSCs, to counteract the mitogenic and profibrotic activities of PECs during renal disease.

## Conclusion

These findings advance our knowledge of the therapeutic potential of distinct stromal cell populations of non-renal and renal origin in experimental progressive nephropathy, pointing to human ucMSCs as the most attractive candidate for promoting renal tissue repair, possibly through the preservation of glomerular structural and functional integrity and by shutting down inflammatory processes. Our data also demonstrate the renoprotective effects of ucMSC-derived conditioned medium, which might be a promising clinical therapeutic tool, overcoming the weaknesses and risks associated with the use of native stromal cells for patients with CKD.

## Additional file

Additional file 1: Supplementary Methods, Table and Figures. (PDF 4792 kb)

## Abbreviations

ADR: Adriamycin
AKI: Acute kidney injury
Ang II: Angiotensin II
bmMSC: Bone marrow-derived mesenchymal stromal cell
BUN: Blood urea nitrogen
CKD: Chronic kidney disease
CM: Conditioned medium
EV: Extracellular vesicle
HNA: Human nuclear antigen
kPSC: Kidney perivascular stromal cell
MSC: Mesenchymal stromal cell
mTOR: Mammalian target of rapamycin
PEC: Parietal epithelial cell
P-H3: Phospho H3-histone
Sesn2: Sestrin 2
TGF-β: Transforming growth factor beta
ucMSC: Umbilical cord-derived mesenchymal stromal cell
