# Supplementary material for: Therapeutic potential of stromal cells of non-renal or renal origin in experimental chronic kidney disease
Source: Stem Cell Res Ther. 2018 Aug 14;9:220. doi: 10.1186/s13287-018-0960-8 (PMC6092807; doi:10.1186/s13287-018-0960-8)
Supplement: Supplementary file 1 — Supplementary Methods, Table and Figures. (PDF 4792 kb) [file 13287_2018_960_MOESM1_ESM.pdf]

Supplementary Methods

Human cell isolation and characterisation

Human bone marrow mesenchymal stromal cells

Human bone marrow mesenchymal stromal cells (bmMSCs) were isolated and characterised as previously described [1]. Briefly, human bone marrow aspirates were collected from adult subjects (30–40 years old). Mononuclear cell fractions were obtained after Ficoll-Hypaque gradient centrifugation and cells were plated at $2x10^5$ cells/cm^2 in αMEM containing 5% human platelet lysate (hPL) from healthy donors, gentamycin 0.1mM and heparin 1000 IU. After 3-4 days non-adherent cells were removed and adherent cells were expanded [1]. Flow cytometric analysis was used to characterise the immunophenotype of bmMSCs, which expressed CD105, CD90 and CD73, while they were negative for CD45, CD34, CD14 and HLA-DR markers. Moreover, bmMSCs showed differentiation potential towards adipocytes, osteoblasts and chondrocytes [1].

Human umbilical cord mesenchymal stromal cells

Human umbilical cord mesenchymal stromal cells (ucMSCs) were collected after either normal vaginal delivery or Caesarean sections, as previously described [2]. Briefly, the UC was cut into 5 cm-long segments and subsequently cut longitudinally to expose the inner surface and transferred to a 150mm Petri dish containing αMEM (Thermo Fisher Scientific Life Science, Waltham, MA, http://www.thermofisher.com) enriched with 5% hPL [2] and gentamycin. After 1 week, remaining UC tissue was removed and adherent cells expanded [2]. The umbilical cord MSC phenotype was analysed by flow cytometry and showed the expression of standard mesenchymal stromal cell markers such as CD73, CD90 and CD105, while ucMSCs were negative for the typical haematopoietic and endothelial cell markers CD31, CD34 and CD45. Moreover, ucMSCs were able to differentiate into osteocytes and chondrocytes, but did not possess adipogenic potential [2].

Human parietal epithelial cells

Human parietal epithelial cells (PECs) were isolated and characterised as previously described [3]. Briefly, PECs were recovered from total renal cells isolated from a normal kidney fragment using CD133-immunomagnetic separation. Clones were generated from CD133+ cells by limiting dilution, and immunofluorescence and FACs analysis were used to identify clones positive for CD24 and CD106 [3]. PECs were grown at 37°C and 5% CO_2, in EGM-MV (Lonza, Basel, Switzerland, http://www.lonza.com) supplemented with 20% FBS Hyclone (FBS HY) (Thermo Fisher Scientific Life Science).

Quantification of human stromal cell engraftment in renal tissue

To identify the presence of human stromal cells in rat renal tissue, anti-human nuclear antigen (HNA) antibody (Merck Millipore, Darmstadt, Germany, http://www.merckmillipore.com) or anti-human mitochondria antibody (hMITO) was used. Briefly, 3-μm thick sections from OCT-included kidney specimens 45 were fixed for 10 minutes in cold acetone at 4°C. Then, sections were incubated with blocking solution, 1% BSA (Sigma-Aldrich, St. Louis, MO, USA, https://www.sigmaaldrich.com) in PBS 1X, for 1 hour followed by anti-HNA-Cy3 conjugate antibody (1:50; clone 235-1, Merck Millipore) or anti-hMITO-Cy3 conjugate antibody (1:50; MAB1273C3, Merck Millipore) at 4°C overnight. Cell nuclei were counterstained with 4',6-diamidino-2-phenylindole (DAPI, Sigma-Aldrich) and the renal structure was marked using FITC-wheat germ agglutinin (WGA) (1:400; Vector Laboratories, Burlingame, CA, USA, https://vectorlabs.com) or Alexa Fluor^{TM}633 conjugate –WGA (Invitrogen). Six sections for each animal were analysed at confocal microscope (Zeiss, Jena, Germany, https://www.zeiss.it), and HNA- or hMITO-positive cells were counted. Data were expressed as number of HNA or hMITO-positive cells/10^5 renal cells.

Other organs as heart, liver and lung were also processed for quantification of non-renal or renal stromal cells positive for HNA (3 sections for each organ, n=3 animals).

Renal Morphology

Kidney samples were fixed in Duboscq-Brazil. Paraffin-embedded sections (3-µm) were stained with periodic acid-Schiff reagent. At least 15-20 glomeruli were examined for each rat, and the extent of lesions was expressed by giving a score from 0 to 4 related to the percentage of glomerular tuft occupied by lesions (0 = no lesions; 1 = lesions affecting ≤25% of the glomerulus; 2 = lesions affecting 26 to 50% of the glomerulus, 3 = lesions affecting 51 to 75% of the glomerulus, 4 = lesions affecting 76 to 100% of the glomerulus). The formation of synechiae was evaluated at 14 days after ADR injection and data were expressed as percentage of glomeruli with different degrees of lesions. The extent of glomerulosclerosis was evaluated at 28 days after ADR, and glomerular damage was expressed as the percentage of glomeruli affected by glomerulosclerosis (%GS).

Immunohistochemistry

For immunofluorescence experiments, sections (3-µm) from OCT or PLP-fixed kidney specimens were analysed as appropriate. After antigen unmasking and blocking of nonspecific sites, sections were incubated with the following primary antibodies: rabbit anti-Wilm´s tumour 1 (WT1, 2 µg/ml, Santa Cruz, CA, USA, https://www.scbt.com), goat anti-nephrin (0.2 µg/ml; Santa Cruz), rabbit anti-claudin 1 (undiluted, Thermo Fisher Scientific Life Science), mouse anti-nestin (1:100; BD Biosciences, Milan, Italy, https://www.bdbiosciences.com), mouse anti-rat endothelial cell antigen (RECA-1; 1:100, R&D System, Minneapolis, MN, USA, https://www.rndsystem.com), rabbit anti-rat fibronectin (1:400, Thermo Fisher Scientific Life Science), mouse anti-rat ED1 (1:100, Merck Millipore), goat anti-CD206 (Santa Cruz) or rabbit anti-cleaved caspase-3 (1.50, Cell Signaling, Denver, MA, USA, https://www.cellsignal.com). Then, the sections were

incubated with the appropriate secondary antibody (Jackson ImmunoResearch Laboratories, West Grove, PA, USA, https://www.jacksonimmuno.com). Slides were stained with 4′,6-diamidino-2-phenylindole (DAPI, Sigma-Aldrich), and those incubated with anti-WT1 antibody were counterstained with FITC-labelled wheat germ agglutinin (WGA) (Vector Laboratories). Double and triple fluorescence labelling was analysed by an inverted confocal laser-scanning microscope (LS 510 Meta; Zeiss), and 10-15 random images/section (n=3 sections), for each animal, were acquired. Fibrosis was evaluated at 28 days, giving a score between 0 to 3, analysing the thickening of fibronectin staining along the Bowman's capsule (BC).

Apoptosis was analyzed quantifying the glomerular cleaved caspase-3 expression, by using the analysis software ImageJ 1.40g. Digitised images were binarised using a threshold for areas of glomerular cleaved caspase-3 staining, and the values were expressed as percentage of area occupied by cleaved caspase-3 staining (n=3 sections for each animal).

TGF-β analysis

Serum TGF-β levels were measured using a commercial available rat ELISA kit (Abcam, Cambridge, U.K., http://www.abcam.com) employing internal controls, as proposed by the manufacturer and analyzed with an ELISA microplate reader at 450 nm. Serum samples were previously acidified to activate latent TGF-β to its immunoreactive form. The limit quantification was 8 pg/ml. The results were calculated in ng/ml by fitting to a standard curve, obtained using human recombinant TGF-β.

Morphometrical analysis

Glomerular podocytes were identified as cells positive for WT1. Estimation of glomerular volume (VG) was performed using a computer-based image analysis system (Mac OS09; Apple Computer) as previously described [4]. Mean value of VG and the estimation of the average

number of podocytes per glomerulus were determined by the stereological method of particle density proposed by Weibel [5].

Glomerular capillary volume density (Vv) was quantified as RECA-1 positive vessels in 15-20 glomeruli per rats randomly acquired (n=3-7 rats for each group). Renal sections were digitalised using an inverted confocal laser microscopy (original magnification, x630; LSM 510 Meta; Carl Zeiss). By using the analysis software ImageJ 1.40g, digitised images were binarised using a threshold for areas of RECA-1 staining, and the values were expressed as percentage of area occupied by RECA-1 on total area of the acquired field.

Immunofluorescence studies in vitro

Proliferation was assessed in PECs fixed with 2% PFA, permeabilized with Triton 0.3% (Sigma-Aldrich) and then incubated with blocking solution composed of PBS 1X, 2%BSA (Sigma-Aldrich), 2%FCS (Thermo Fisher Scientific Life Science), 0.2% bovine gelatine (Sigma-Aldrich). Subsequently, PECs were incubated at 4°C overnight with rabbit anti-phospho-Histone H3 (Ser10) antibody (anti P-H3 antibody, 1:75; Cell Signalling) followed by secondary antibody FITC goat anti-rabbit (1:100; Jackson ImmunoResearch Laboratories). Nuclei were counterstained with DAPI (Sigma-Aldrich). Ten fields/samples were randomly acquired using confocal microscopy (Zeiss). The number of P-H3-positive cells was counted, normalised for the number of DAPI-positive cells and expressed as the percentage of P-H3-positive PECs per total of PECs in each high-power field (HPF).

To evaluate sestrin 2, claudin1 or fibronectin expressions, fixed PECs were incubated with rabbit anti-sestrin 2 antibody (1:100, ProteinTech Group, Chicago, IL, USA, https://www.ptglab.com), with rabbit anti-claudin 1 antibody (undiluted, Thermo Fisher Scientific Life Science) or with rabbit anti-fibronectin antibody (1:100; Thermo Fisher Scientific Life Science), respectively. Donkey or goat anti-rabbit Cy3 (1:50; Jackson ImmunoResearch Laboratories) were used as appropriate secondary antibodies. Cell nuclei were stained with DAPI (Sigma-Aldrich). Ten fields

for each sample were acquired using confocal microscopy (magnification 40X) (Zeiss). ImageJ was used to evaluate the mean fluorescent intensity (MFI) that was normalised for the number of DAPI-positive cell nuclei (MFI/cell).

References

1. Capelli C, Domenghini M, Borleri G, Bellavita P, Poma R, Carobbio A, et al. Human platelet lysate allows expansion and clinical grade production of mesenchymal stromal cells from small samples of bone marrow aspirates or marrow filter washouts. Bone Marrow Transplant. 2007;40:785–91.

2. Capelli C, Gotti E, Morigi M, Rota C, Weng L, Dazzi F, et al. Minimally manipulated whole human umbilical cord is a rich source of clinical-grade human mesenchymal stromal cells expanded in human platelet lysate. Cytotherapy. 2011;13:786–801.

3. Morigi M, Locatelli M, Rota C, Buelli S, Corna D, Rizzo P, et al. A previously unrecognized role of C3a in proteinuric progressive nephropathy. Sci Rep. 2016;6:28445.

4. Macconi D, Bonomelli M, Benigni A, Plati T, Sangalli F, Longaretti L, et al. Pathophysiologic implications of reduced podocyte number in a rat model of progressive glomerular injury. Am J Pathol. 2006;168:42–54.

5. Wiggins RC. The spectrum of podocytopathies: a unifying view of glomerular diseases. Kidney Int. 2007;71:1205–14.

Table S1 Renal function in Control and Adriamycin (ADR) rats receiving saline, bmMSCs, ucMSCs, kPSCs or CM-ucMSCs.

	Blood urea nitrogen (mg/dl)
	**28 days**
**Control**	20.22±0.29
**ADR+saline**	27.35±1.21
**ADR+bmMSCs**	25.17±1.72
**ADR+ucMSCs**	24.30±1.87
**ADR+kPSCs**	25.95±1.68
**ADR+CM-ucMSCs**	27.85±3.64

Data are mean±SE.
bmMSCs: bone marrow mesenchymal stromal cells
ucMSCs: umbilical cord mesenchymal sromal cells
kPSCs: kidney perivascular stromal cells
CM-ucMSCs: conditioned medium obtained from umbilical cord mesenchymal stromal cells

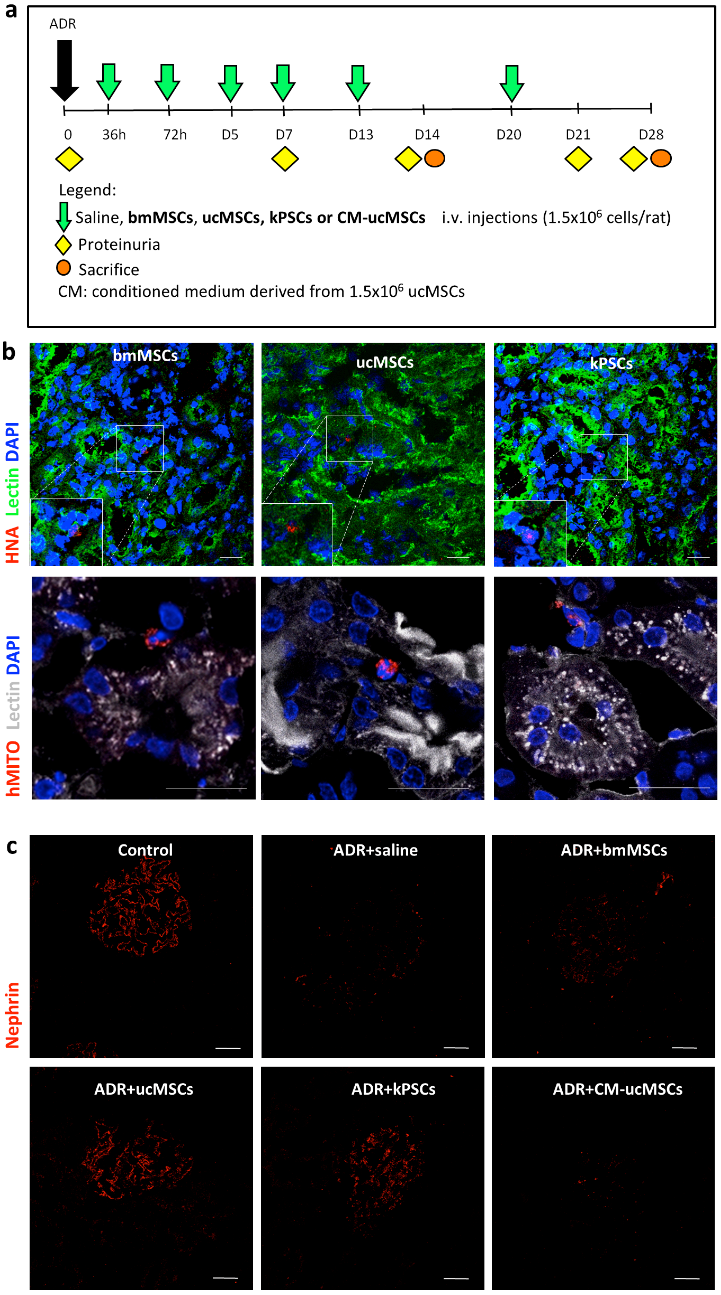

Fig. S1 (a) Experimental design of in-vivo setting. Rats received intravenous injection (i.v.) of ADR (7.9 mg/Kg) and starting from 36 hours were injected i.v. with different populations of stromal cells or CM-ucMSCs. (b) Representative micrographs of kidney tissue from ADR rats showing the engraftment at 14 days of human bmMSCs, ucMSCs or kPSCs labelled with anti-human nuclear antigen (HNA, upper panels) or anti-human mitochondria (hMITO, red, lower panels) antibody. Renal structures were stained with lectin (green or white) and nuclei with DAPI (blue). Scale bar 20 µm. (c) Representative micrographs of renal sections from control and ADR-rats receiving saline, bmMSCs, ucMSCs, kPSCs or CM-ucMSCs at 14 days showing nephrin expression. Scale bar 20 µm. ADR adriamycin, bmMSC bone marrow-derived mesenchymal stromal cell, CM-ucMSC conditioned medium obtained from umbilical cord-derived mesenchymal stromal cell, D day, DAPI 4′,6-diamidino-2-phenylindole, kPSC kidney perivascular stromal cell.

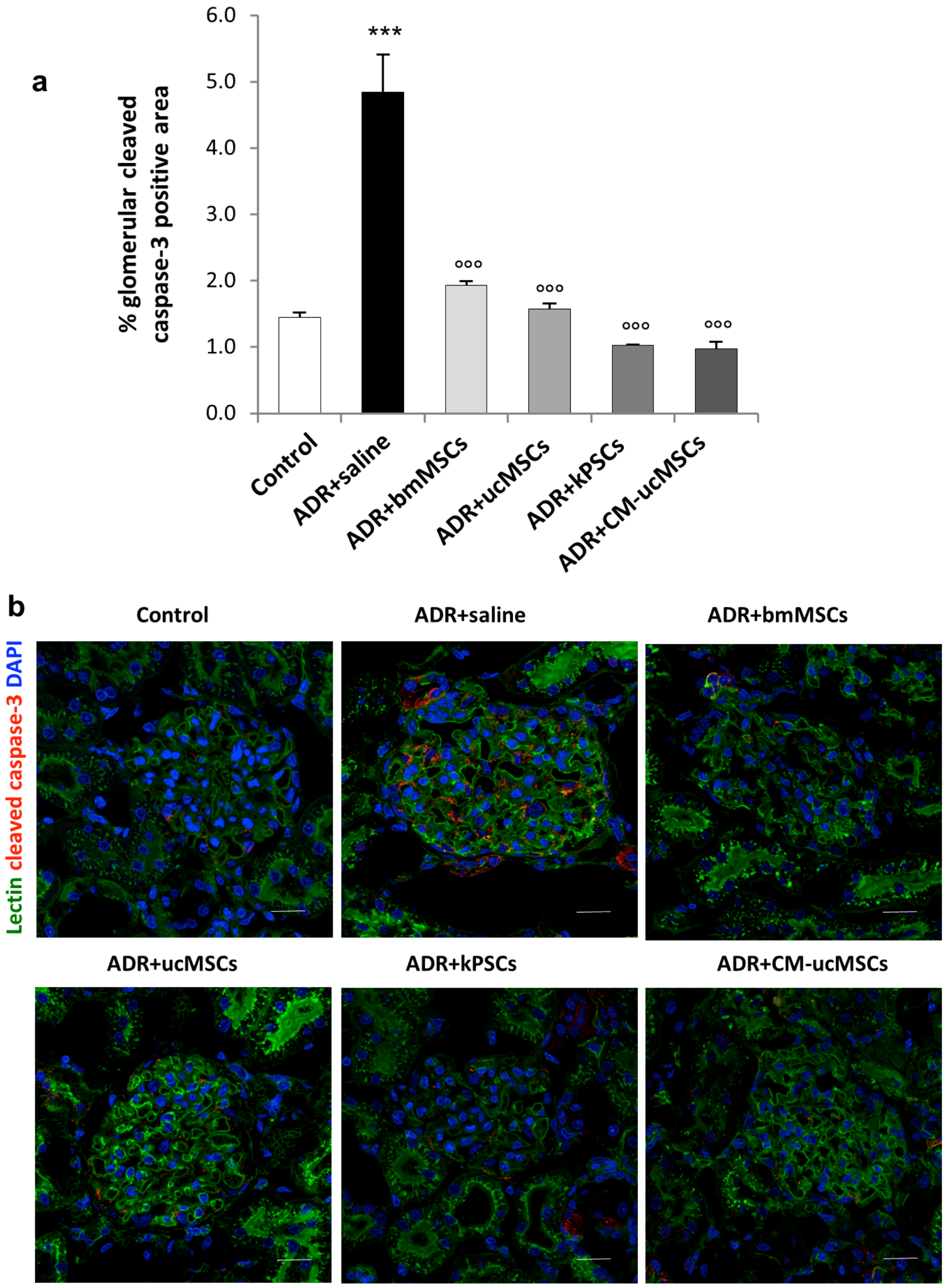

Fig. S2 (a) Quantification of apoptosis, assessed as percentage of cleaved caspase-3 positive area in glomeruli of control and ADR rats receiving saline, bmMSCs, ucMSCs, kPSCs or CM-ucMSCs at 14 days. Data are mean ± SE. ***p<0.001 vs. control, °°°p<0.001 vs. ADR+saline. (b) Representative micrographs of renal sections from control and ADR rats receiving saline, bmMSCs, ucMSCs, kPSCs or CM-ucMSCs at 14 days showing cleaved-caspase 3 expression (red). Renal structures were stained with lectin (green) and nuclei with DAPI (blue). Scale bar 20 µm. ADR adriamycin, bmMSC bone marrow-derived mesenchymal stromal cell, CM-ucMSC conditioned medium obtained from umbilical cord-derived mesenchymal stromal cell, DAPI 4′,6-diamidino-2-phenylindole, kPSC kidney perivascular stromal cell.
